# Preliminary analysis of different tools in emotional competence assessment in patients with schizotypal disorder

**DOI:** 10.1192/j.eurpsy.2021.1403

**Published:** 2021-08-13

**Authors:** M. Vinogradova, O. Khokhlova, I. Oleichik, A. Ermusheva, N. Salikhianova

**Affiliations:** 1 Department Of Neuro- And Pathopsychology, Faculty Of Psychology, Lomonosov Moscow State University, Moscow, Russian Federation; 2 Psychology Department, Middlesex University Dubai, Dubai, United Arab Emirates; 3 Clinical Department Of Endogenous Mental Disorders And Affective States, Mental Health Research Centre, Moscow, Russian Federation; 4 Department Of Pedagogy And Medical Psychology, I.M. Sechenov First Moscow State Medical University (Sechenov University), Moscow, Russian Federation; 5 Department Of Rehabilitation, Mental-health clinic No.1 named after N.A. Alexeev, Moscow, Russian Federation

**Keywords:** emotional competence, empathy, schizotypal disorder, social cognition

## Abstract

**Introduction:**

Dysfunctional emotional competence is known as one of the key characteristics of patients with schizotypal disorder. Methods that aim to assess this problem are differently organized and might elicit these deficits differently. Therefore, there is a need in better understanding of scope of problems that might be assessed using different tools in patients with schizotypal disorder.

**Objectives:**

To examine the differences in affective empathy and perception of emotions in normal subjects and patients with schizotypal disorder.

**Methods:**

The sample consisted of 14 patients with schizotypal disorder (F21) (M=19.07, SD=3.17) and 53 healthy individuals (M=22.98, SD=2.77) with equal educational level. Participants were given Affective Responsiveness Test (AR) and Emotional Perspective Taking (ERT) (Derntl et al, 2009) and “Reading the Mind in the Eyes” (RME) Test (Baron-Cohen et al., 2001).

**Results:**

There were significant differences in accuracy of ERT performance between patients with schizotypal disorder (M=80.64, SD=8.17) and healthy individuals (M = 86.62, SD = 8.67), t (65) = -2.32, p = .023. Patients were also found to give less correct answers than healthy controls while carrying out AR, and to need more time for both tasks. However, these differences were not statistically significant. Surprisingly, no significant differences were found for perception of emotions (RME) test, although patients in general gave less correct answers.

**Conclusions:**

It might be assumed that EPT is the most sensitive tool in assessing emotional deficits in patients with schizotypal disorders. Further research is needed to understand the possible reasons for other tests not showing significant results.

